# Predictive factors for postoperative outcomes after reverse shoulder arthroplasty: a systematic review

**DOI:** 10.1186/s12891-024-07500-3

**Published:** 2024-06-04

**Authors:** J. R. W. Crutsen, F. O. Lambers Heerspink, E. A. P. van Leent, E. R. C. Janssen

**Affiliations:** 1 Department of Orthopaedic Surgery, VieCuri Medical Centre, Tegelseweg 210, Venlo, 5912 BL The Netherlands; 2 IQ Healthcare, Radboud University Medical Centre, Nijmegen, The Netherlands

**Keywords:** Shoulder arthroplasty, Reverse shoulder arthroplasty, Predictive outcomes, Prognostic factors, Postoperative outcomes

## Abstract

**Background:**

The use of reverse total shoulder arthroplasty (RTSA) has increased at a greater rate than other shoulder procedures. In general, clinical and functional outcomes after RTSA have been favorable regardless of indication. However, little evidence exists regarding patient specific factors associated with clinical improvement after RTSA. Predicting postoperative outcomes after RTSA may support patients and physicians to establish more accurate patient expectations and contribute in treatment decisions. The aim of this study was to determine predictive factors for postoperative outcomes after RTSA for patients with degenerative shoulder disorders.

**Methods:**

EMBASE, PubMed, Cochrane Library and PEDro were searched to identify cohort studies reporting on predictive factors for postoperative outcomes after RTSA. Authors independently screened publications on eligibility. Risk of bias for each publication was assessed using the QUIPS tool. A qualitative description of the results was given. The GRADE framework was used to establish the quality of evidence.

**Results:**

A total of 1986 references were found of which 11 relevant articles were included in the analysis. Risk of bias was assessed as low (*N* = 7, 63.6%) or moderate (*N* = 4, 36.4%). According to the evidence synthesis there was moderate-quality evidence indicating that greater height predicts better postoperative shoulder function, and greater preoperative range of motion (ROM) predicts increased postoperative ROM following.

**Conclusion:**

Preoperative predictive factors that may predict postoperative outcomes are: patient height and preoperative range of motion. These factors should be considered in the preoperative decision making for a RTSA, and can potentially be used to aid in preoperative decision making.

**Level of evidence:**

Level I; Systematic review.

**Supplementary Information:**

The online version contains supplementary material available at 10.1186/s12891-024-07500-3.

## Introduction

The use of reverse total shoulder arthroplasty (RTSA) has increased at a greater rate than other shoulder arthroplasty procedures [1, 2]. This trend can be attributed to an aging population that desires to remain physically active, as well as the expanding range of indications for RTSA [3]. The most common indication for RTSA is rotator cuff arthropathy, but also includes several conditions that were challenging to treat with an anatomical shoulder arthroplasty (TSA), such as glenohumeral arthritis with deformity of the glenoid, proximal humerus fracture, failed shoulder arthroplasty and tumors [4].

In general, RTSA has shown favorable clinical and functional outcomes regardless of indication [3]. Lindbloom et al. reported significant improvement in all clinical outcome scores [5]. However, some patients may experience better results after RTSA than others, as outcomes can be affected by several factors, such as the underlying etiology for glenohumeral degeneration, comorbidities, demographics, pre-operative pain, and daily functioning [1, 6]. However studies show conflicting results. Poor outcomes after RTSA have been noted in certain small subgroups of patients, causing some concern. It is important to ensure that costly and burdensome arthroplasty procedures are only performed on patients who are likely to benefit from the procedure [3].

Despite the growing use of RTSA, there is limited evidence available regarding patient specific factors associated with postoperative improvement after RTSA [1]. Prediction models can be used to estimate postoperative outcomes after RTSA and may facilitate patients and physicians in making well informed treatment decisions [1]. In different orthopedic populations nomograms have been developed, based on preoperative predictive factors, to predict individual post-operative success chance of a patient [7–9]. For example, using the nomogram for spinal fusion, the chance of achieving a clinically relevant postoperative pain reduction is predicted between 0 and 100% [7]. Based on this percentage, patient and surgeon can engage in well-informed decision making if spinal surgery is worthwhile. Using such prediction models, may lead to better patient selection before orthopedic surgery and greater patient satisfaction after surgery [6].

Treatment success in RTSA is measured using a multitude of outcomes, such as patient-reported outcome measures (PROMs) (e.g., functional recovery, pain) and clinical outcomes (e.g., complication rate, failure rate). These outcomes can also be taking into account in prediction models.

Therefore, we performed a systematic review evaluating associations between preoperative predictive factors and postoperative outcomes (PROMs and clinical outcomes) after RTSA. The aim of this study was to identify predictive factors that are predictive for postoperative outcomes after RTSA.

## Material and methods

The study protocol of this systematic review was registered in PROSPERO (CRD42021235388). We reported our systematic review according to the Preferred Reporting Items for Systematic Review and Meta-Analyses (PRISMA) guidelines. In this article some terminology on prediction modelling research is used. Some important recurrent terms are clarified in Table 1.

**Table 1:** Terminology and definitions on prediction modelling

**Measures of association:** a wide variety of statistics that quantify the strength and direction of the relationship between exposure and outcome variables, enabling comparison between different groups [10]
**Prognostic factor**: a measurement that is associated with clinical outcome in the absence of therapy or with the application of a standard therapy that patients are likely to receive [11]
**Prediction model:** these models use multiple prognostic factors in combination to predict the risk of future clinical outcomes in individual patients [12]

### Literature search

We conducted a comprehensive search of various data sources including EMBASE, PubMed, Cochrane Library and PEDro to identify relevant studies reporting predictive factors for RTSA before the first of February 2024, using the keywords: treatment outcome; prognosis; prediction; shoulder; arthroplasty; prosthesis; and reversed (Appendix 1). No restrictions for date or language were used. The reference lists of eligible studies were manually scanned for potentially relevant papers.

### Study selection

The results of the literature search were collected in the reference management program Endnote (Clarivate Analytics, version 9.3.3). Duplicates were identified by one author (JC). Two independent reviewers (JC and EvL) screened all articles on title and abstract to determine their eligibility. In case of disagreement, consensus was achieved via a consensus meeting. Thereafter, full-text screening was conducted by the same reviewers. Duplicates and articles of which the full-text was unavailable were excluded at this stage. The following inclusion criteria were used: 1) RTSA (procedure); 2) Pro- or retrospective cohort study (design); 3) explore which predictive factors independently contribute to the prediction of an outcome, i.e. some type of association measure. The following association measures were eligible: relative risks, odds ratios, risk difference, regression coefficients, correlation/prediction coefficients, their 95% confidence intervals and *P*-values. We excluded studies in which patients with revisions, tumors, or fractures were included. Studies that were identified as sub-studies of included studies were used to complete outcome measures if these were not reported in the publication of the main study.

### Risk of bias of included studies

The methodologic validity of included studies was assessed by two independent researchers (JC and EJ) using the Quality In Prognosis Studies (QUIPS) tool [13]. The risk of bias for individual studies was considered as: 1) low, if all domains were rated as low-moderate risk of bias; 2) moderate, when one domain was rated as high and the remaining domains were rated as low-moderate; 3) or high, when more than one domain was rated as high risk of bias. Conflicts in grading were resolved in a consensus meeting.

### Data extraction

One researcher (JC) extracted all data using a customized template in Excel (Microsoft, version 16.43). Prior to analysis, all extracted data were checked with source articles to confirm accuracy by two researchers (JC and EJ). Differences were resolved in a consensus meeting. If data were missing, a maximum of two attempts were made to contact the corresponding author to retrieve the missing data. The extracted data on predictive factors were organized in groups of predictive factors: 1) Personal factors: age, sex, height, and surgery on dominant side; 2) Disorders: diagnoses, prior shoulder surgery, and comorbidities; 3) Function: preoperative range of motion (ROM), preoperative American Shoulder and Elbow Shoulder (ASES) score, and preoperative visual analog scale (VAS) score. The extracted data included data on: 1) the authors; 2) year of publication; 3) follow up duration; 4) preoperative predictive factor(s); 5) association measure, including *p*-values and confidence intervals; 6) postoperative outcomes measure.

### Narrative evidence synthesis

The narrative evidence synthesis was performed and included a tabulation of results to facilitate comparison between studies, with patterns of predictions and similarities/differences between studies identified and discussed. A meta-analysis was not feasible due to the heterogeneity in reported outcome measures.

### GRADE assessment

The GRADE PH was used to assess the quality of evidence for each outcome in relation to the potential predictive factor [14]. The GRADE PH asses seven different factors for quality of the evidence: I) study phase; II) study limitations, as assessed with the QUIPS; III) inconsistency; IV) indirectness; V) publication bias and; VI) effect size. Moreover, quality of evidence can be upgraded if there is evidence of a dose effect relationship.

## Results

### Literature search

**Fig. 1:**
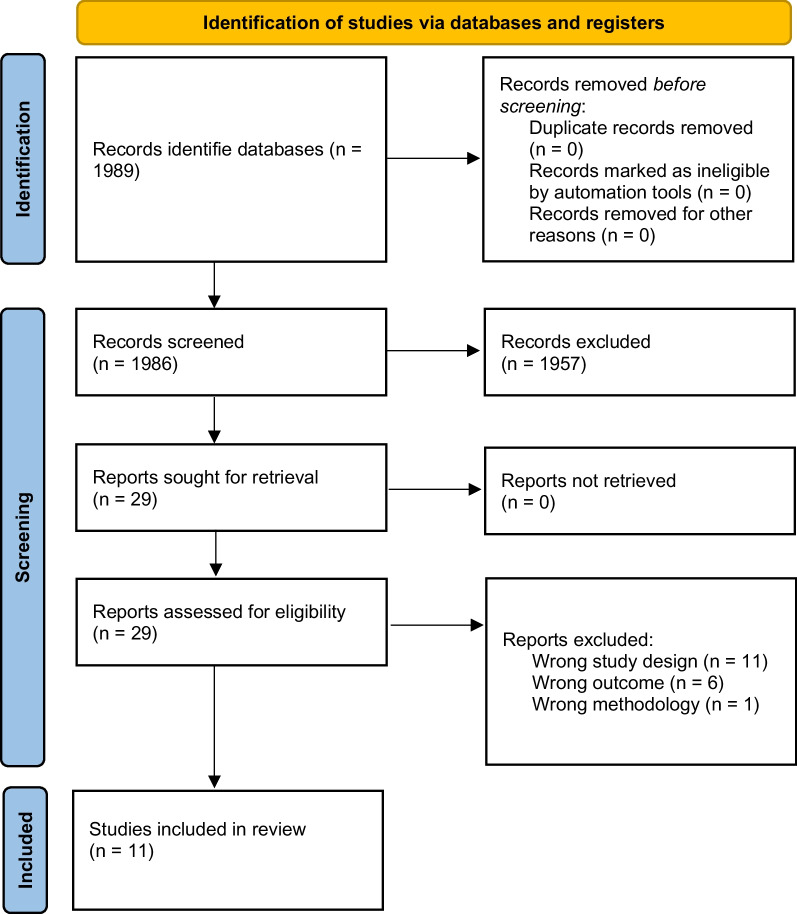
PRISMA flowchart

The search strategy identified 1986 potentially relevant articles. The abstracts of these studies were reviewed to determine the eligibility (Fig. 1). We included 29 articles for full-text screening. Eighteen studies were excluded after full-text screening, based on study design (*n* = 11), incorrect outcome measures (*n* = 5), or lack of predictive data (*n* = 1). Eleven studies were included in the qualitative analysis. According to the QUIPS tool 36.4% (*n* = 4) of the articles were classified as moderate risk of bias and 63.6% (*n* = 7) of the articles were classified as low risk of bias (Table 2). The extracted data of the included studies were summarized in Table 3. Due to the heterogeneity between studies with regards to reported outcome measures and the timing of follow-up measurement we were unable to pool any of the predictive values.

**Table 2:** Assessment of methodologic quality

Included study	Study participation	Study attrition	Predictive factor measurement	Outcome measurement	Study confounding	Statistical analysis and reporting	Overall conclusion
Friedman, R. J., et al. (2018) [1]	Low risk	Moderate risk	Low risk	Low risk	High risk	Low risk	Moderate risk
Werner, B. C., et al. (2016) [3]	Low risk	Moderate risk	Moderate risk	Low risk	Moderate risk	Low risk	Moderate risk
Rauck, R. C., et al. (2018) [15]	Low risk	Low risk	Low risk	Low risk	Low risk	Low risk	Low risk
Schwartz, D. G., et al. (2014) [16]	Low risk	High risk	Low risk	Low risk	Low risk	Moderate risk	Moderate risk
Collin, P., et al. (2017) [17]	Low risk	Low risk	Low risk	Low risk	Low risk	Low risk	Low risk
DeVito, P., et al. (2019) [18]	Low risk	High risk	Low risk	Low risk	Low risk	Moderate risk	Moderate risk
Rauck, R. C., et al. (2020) [19]	Low risk	Low risk	Low risk	Low risk	Low risk	Moderate risk	Low risk
Carducci, M. P., et al. (2019) [20]	Low risk	Moderate risk	Low risk	Low risk	Low risk	Low risk	Low risk
Friedman, R. J., et al. (2019) [21]	Low risk	Moderate risk	Low risk	Low risk	Low risk	Low risk	Low risk
Morris, B. J., et al. (2015) [22]	Low risk	Moderate risk	Low risk	Low risk	Low risk	Low risk	Low risk
Baram, A., et al. (2020) [23]	Moderate risk	Moderate risk	Low risk	Low risk	Low risk	Low risk	Low risk

For each of the 6 domains in the QUIPS tool, responses to the prompting items are taken together to inform the judgment of risk of bias. To grade the tool, each of the 6 potential bias domains is rated as having high, moderate, or low risk of bias. The risk of bias for individual studies was considered as: low if all domains were rated as low-moderate risk of bias; moderate when one domain was rated as high and the remaining domains were rated as low-moderate; or high when more than one domain was rated as high risk of bias

**Table 3:** Overview of results

Predictive factor	Article	Outcome	Follow-up	Association	Interpretation
	**Personal factors**				
**Age**	Friedman, R.J., et al. (2018) [1] (Moderate risk of bias)	ASES	At least 6 weeks	Mixed linear model* Coefficient: 0.19 95% CI: 0.04–0.34 *P*-value: 0.011	Increasing age was associated with better postoperative ASES score *Sex
		SPADI	At least 6 weeks	Mixed linear model* Coefficient: -0.29 95% CI: -0.46–0.07 *P*-value: 0.020	Increasing age was associated with better postoperative SPADI score *Sex
		ROM (measured by the active abduction and forward flexion)	At least 6 weeks	Mixed linear model* Active abduction Coefficient: -0.26° 95% CI: -0.46°-0.07° *P*-value: 0.007 Forward flexion Coefficient: -0.39° 95% CI: -0.61°-0.16° *P*-value: 0.001	Increasing age was associated with decreased postoperative ROM (active abduction and forward flexion) *Sex
	Friedman, R.J., et al. (2019) [21] (Low risk of bias)	ROM (measured by the active abduction and forward flexion)	Mean 49 months	Linear regression Active abduction Coefficient: -0.29 *P*-value: 0.024 Forward flexion Coefficient: -0.38 *P*-value: < 0.001	Increasing age was associated with a decrease in postoperative ROM (active abduction and forward flexion)
	Morris, B.J., et al. (2015) (Low risk of bias)	Infection rate	Min. 1 year	Bivariate logistic regression OR: 0.95 95% CI: 0.91–0.99 *P*-value 0.012 Multivariable logistic regression* OR: 0.95 95% CI: 0.91–0.99 *P*-value: 0.021	Increasing age was associated with an increased risk for periprosthetic infection *Failed arthroplasty
**Sex**	Werner, B.C., et al. (2016) [3] (Moderate risk of bias)	Failure (Defined as poor postoperative improvement described as change in ASES score less than 1 SD below average)	Min. 2 years	Logistic regression OR: 7.9 95% CI: 1.9–31.7 *P*-value: 0.004	Male sex was associated with poor postoperative improvement
	Schwartz, D.G., et al. (2014) [16] (Moderate risk of bias)	ROM (measured by the forward flexion)	Range 1–3 years	Multivariable regression* Coefficient: 8.26 95% CI: 1.80–14.72 *P*-value: 0.012	Male sex was associated with better postoperative ROM (forward flexion) *Preoperative FF + *Intraoperative FF*
	DeVito, P., et al. (2019) [18] (Moderate risk of bias)	SST	Min. 2 years	Univariate logistic regression OR: 2.431 95% CI: 1.297–4.226 *P*-value 0.005	Not specified which sex is associated with better postoperative SST scores
	Baram, A. et al. (2020) [23] (Low risk of bias)	Revision risk	1 year	Cox regression Hazard ratio: 2.74 95% CI: 1.59–4.73 *P*-value: < 0.01	Male sex was associated with an increased risk of revision
	Friedman, R.J., et al. (2019) [21] (Low risk of bias)	ROM (measured by the active external rotation and active internal rotation)	Mean 49 months	Linear regression Active external rotation Coefficient: -4.38 *P*-value: 0.014 Active internal rotation Coefficient: -0.32 *P*-value: 0.034	Male sex was associated with a decreased postoperative ROM (active external rotation and active internal rotation)
**Height**	DeVito, P., et al. (2019) [18] (Moderate risk of bias)	SST	Min. 2 years	Univariate logistic regression OR: 1.038 95% CI: 1.009–1.069 *P*-value 0.010	Greater height was associated with an increased postoperative SST score
	Friedman, R.J., et al. (2019) [21] (Low risk of bias)	ASES	Mean 49 months	Linear regression Coefficient: 0.39 *P*-value: 0.007	Greater height was associated with an increased in postoperative ASES score
		ROM (measured by the active external rotation and active internal rotation)	Mean 49 months	Linear regression Active external rotation Coefficient: 0.42 *P*-value: 0.032 Active internal rotation Coefficient: 0.04 *P*-value: 0.015	Greater height was associated with an increased in postoperative ROM (active external rotation and active internal rotation)
		Shoulder function score	Mean 49 months	Linear regression Coefficient: 0.04 *P*-value: 0.031	Greater height was associated with an increased in postoperative shoulder function score
		SST score		Linear regression Coefficient: 0.09 *P*-value: 0 < .001	Greater height was associated with an increased postoperative SST score
**Dominant arm**	Collin, P. et al. (2017) [17] (Low risk of bias)	ROM (measured by the active forward flexion)	6 weeks	Multivariate logistic regression* OR: 0.115 95% CI: 0.0182–0.0725 *P*-value 0.0213	Surgery on the dominant side was associated with an increased postoperative ROM (active forward flexion) *Poor strength of deltoid + *Preoperative SSV* + *Preoperative active AFF*
	DeVito, P., et al. (2019) [18] (Moderate risk of bias)	SST	Min. 2 years	Univariate logistic regression OR: 0.408 95% CI: 0.220–0.757 *P*-value 0.005 Multivariate logistic regression* OR: 0.347 95% CI: 0.177–0.678 *P*-value: 0.002	Surgery on the dominant side was associated with an increased postoperative SST score *Height + *Sex* + *Preoperative VAS pain*
	**Disorders**				
**Diagnosis (Rotator cuff arthropathy)**	Werner, B.C., et al. (2016) [3] (Moderate risk of bias)	Failure (Defined as poor postoperative improvement described as change in ASES score less than 1 SD below average)	Min. 2 years	Logistic regression OR: 4.8 96% CI: 1.2–18.8 *P*-value 0.025	Presence of rotator cuff arthropathy was associated with an increased postoperative improvement
	DeVito, P., et al. (2019) [18] (Moderate risk of bias)	ASES	Min. 2 years	Univariate logistic regression OR: 2.08 95% CI: 1.130–3.829 *P*-value: 0.019 Multivariate logistic regression* OR: 2.018 95% CI: 1.062–3.834 *P*-value: 0.032	Presence of rotator cuff arthropathy was associated with an increased postoperative ASES score *Preoperative VAS pain + *Preoperative ASES total*
	Carducci, M.P., et al. (2019) [20] (Low risk of bias)	Improvement (low pre-to-postoperative ASES score improvement)	Min. 2 years	Multivariate logistic regression* OR: 2.42 95% CI: 0.67–8.83 *P*-value: < 0.05	Presence of rotator cuff arthropathy was associated with an decreased postoperative improvement *Preoperative ASES score + *Preoperative narcotic use* + *Prior shoulder surgery*
**Prior shoulder surgery**	Carducci, M.P., et al. (2019) [20] (Low risk of bias)	ASES (bottom 30th percentile)	Min. 2 years	Multivariate logistic regression* OR: 2.46 95% CI: 1.03–5.83 *P*-value: < 0.05	Prior shoulder surgery was associated with an low postoperative ASES score *Preoperative ASES score + *Primary diagnosis* + *Preoperative narcotic use*
		Improvement (low pre-to-postoperative ASES score improvement)		Multivariate logistic regression* OR: 3.77 95% CI: 1.34–11.23 *P*-value: < 0.05	Prior shoulder surgery was associated with an low postoperative improvement in ASES score *Preoperative ASES score + *Primary diagnosis* + *Preoperative narcotic use*
	Friedman, R.J., et al. (2019) [21] (Low risk of bias)	ROM (measured by the forward flexion)	Mean 49 months	Linear regression Coefficient: -7.05 *P*-value < 0.001	Prior shoulder surgery was associated with a lower postoperative ROM (forward flexion)
		ASES	Mean 49 months	Linear regression Coefficient: -3.32 *P*-value: 0.014	Prior shoulder surgery was associated with a lower postoperative ASES score
**Comorbidities**	Werner, B.C., et al. (2016) [3] (Moderate risk of bias)	Failure (Defined as poor postoperative improvement described as change in ASES score less than 1 SD below average)	Min. 2 years	Continuous variable *P*-value: 0.035	More comorbidities were associated with poor postoperative improvement
	Friedman, R.J., et al. (2019) [21] (Low risk of bias)	ROM (measured by the forward flexion)	Mean 49 months	Linear regression Coefficient: 5.38 *P*-value: 0.031	Hypertension was associated with a better postoperative ROM (forward flexion)
	**Function & Activity**				
**Pre-operative ASES**	Werner, B.C., et al. (2016) [3] (Moderate risk of bias)	Failure (Defined as poor postoperative improvement described as change in ASES score less than 1 SD below average)	Min. 2 years	Continuous variable *P*-value: < 0.001	A higher baseline ASES score was associated with poor postoperative improvement
	DeVito, P., et al. (2019) [18] (Moderate risk of bias)	ASES	Min. 2 years	Univariate logistic regression OR: 0.967 95% CI: 0.950–0.986 *P*-value: < 0.001	A higher baseline ASES score was associated with a better postoperative ASES score
	Carducci, M.P., et al. (2019) [20] (Low risk of bias)	Improvement (low pre-to-postoperative ASES score improvement)	Min. 2 years	Multivariate logistic regression* OR 2.96 95% CI: 2.04–4.62 *P*-value: < 0.05	A higher baseline ASES score was associated with poor postoperative improvement * Primary diagnosis + *Preoperative narcotic use* + *Prior shoulder surgery*
	Friedman, R.J., et al. (2019) [21] (Low risk of bias)	ASES	Mean 49 months	Linear regression Coefficient: 0.32 *P*-value: < 0.001	A higher baseline ASES score was associated with better postoperative ASES score
		ROM (measured by the active external rotation and active internal rotation)	Mean 49 months	Linear regression Active external rotation Coefficient: -0.15 *P*-value: 0.033 Active internal rotation Coefficient: 0.02 *P*-value: < 0.001	A higher baseline ASES score was associated with better postoperative active internal rotation, but lower postoperative active external rotation
	Rauck, R.C., et al. (2020) (Low risk of bias)	Satisfaction (measured by PREMs)	2 years	Poisson regression* Coefficient: -0.010 IRR: 0.99 *P*-value: 0.005	A higher baseline ASES score was associated with a decreased postoperative satisfaction *Age + *Sex* + *Preoperative ROM*
**Pre-operative ROM**	Schwartz, D.G., et al. (2014) [16] (Moderate risk of bias)	ROM (measured by the forward flexion)	Range 1–3 years	Multivariable regression* Coefficient: 0.20 95% CI: 0.11–0.29 *P*-value: < 0.001	Greater forward flexion was associated with better postoperative ROM (forward flexion) *Male sex + *Intraoperative FF*
	Collin, P. et al. (2017) [17] (Low risk of bias)	ROM (measured by the active forward flexion)	6 weeks	Multivariate logistic regression* OR: 15.6 95% CI: 2.92–82.9 *P*-value: 0.00129	Greater forward flexion was associated with better postoperative ROM (active forward flexion) *Non-dominant side injured + *Poor strength of deltoid* + *Preoperative SSV*
	Friedman, R.J., et al. (2019) [21] (Low risk of bias)	ASES	Mean 49 months	Linear regression Active external rotation Coefficient: 0.10 *P*-value: 0.036	Greater ROM (active external rotation and passive external rotation) was associated with better postoperative ASES score
		ROM (measured by abduction, forward flexion and active external rotation)	Mean 49 months	Linear regression Abduction Coefficient: 0.19 *P*-value: < 0.001 Forward flexion Coefficient: 0.22 *P*-value: 0.036 Active external rotation Coefficient: 0.021 *P*-value: < 0.001	Greater ROM was associated with better postoperative ROM: - Better abduction results in better postoperative abduction, but lower postoperative forward flexion - Better forward flexion results in better postoperative forward flexion - Better active external rotation results in better abduction, forward flexion and active external rotation
**Pre-operative pain**	DeVito, P., et al. (2019) [18] (Moderate risk of bias)	SST	Min. 2 years	Univariate logistic regression OR: 1.115 95% CI: 1.002–1.24 *P*-value 0.046 Multivariate logistic regression* OR: 1.151 95% CI: 1.021–1.297 *P*-value 0.021	A higher baseline VAS score was associated with a better postoperative SST score *Surgery on dominant hand + *Height* + *Sex*
		ASES		Univariate logistic regression OR: 1.265 95% CI: 1.113–1.438 *P*-value: < 0.001	A higher baseline VAS score was associated with a better postoperative ASES score
	Friedman, R.J., et al. (2019) [21] (Low risk of bias)	VAS pain score	Mean 49 months	Linear regression Coefficient: 0.16 *P*-value: < 0.001	A higher baseline VAS score was associated with a better postoperative VAS score (less postoperative pain)
		ROM (measured by the forward flexion)	Mean 49 months	Linear regression Coefficient: -1.42 *P*-value: < 0.001	A higher baseline VAS score was associated with a lower postoperative ROM (forward flexion)
**Opioid use**	Carducci, M.P., et al. (2019) [20] (Low risk of bias)	ASES (bottom 30th percentile)	Min. 2 years	Multivariate logistic regression* OR: 3.71 95% CI: 1.18–12.19 *P*-value: < 0.05	Greater opioid use was associated with a decreased postoperative ASES score *Preoperative ASES score + *Primary diagnosis* + *Prior shoulder surgery*
		Improvement (low pre-to-postoperative ASES score improvement)	Min. 2 years	Multivariate logistic regression* OR: 3.38 95% CI: 0.90–12.75 *P*-value: < 0.05	Greater opioid use was associated with a lower postoperative improvement *Preoperative ASES score + *Primary diagnosis* + *Prior shoulder surgery*

*Indicating of all parameters included in the analysis

### Personal factors

#### Age

Two studies with low to moderate risk of bias evaluated the influence of age on shoulder function (ROM) [1, 21] and patient experienced shoulder function (ASES- and SPADI score) [1] (Table 3). In the study by Friedman et al. higher age was associated with better postoperative ASES and SPADI scores after RTSA [1]. Conversely, higher age was associated with a decreased postoperative ROM (measured by the active forward flexion and active abduction) in the same as well as in a different study by Friedman et al. [1, 21]. *According to our evidence synthesis, there is low quality evidence that a higher age has an negative influence on ROM following RTSA, and very low quality evidence suggests that higher age leads to better experienced shoulder function.*

#### Sex

Five studies with low to moderate risk of bias evaluated the influence of sex on shoulder function (ROM) [16, 21] and patient experienced shoulder function (ASES- and SST score) [3, 18]. The majority of the results showed that the male sex is associated with lower postoperative improvement measured using the ASES- and SST score (Table 3) [3, 21, 23]. Concerning postoperative ROM Schwartz et al. and Friedman et al. showed conflicting results [16, 21]. One study presented better postoperative ROM (measured by forward flexion) for the male sex, while the other presented worse postoperative ROM (measured by active external and internal rotation) for the male sex after RTSA [16, 21]. *According to our evidence synthesis, there is low to very low quality evidence that suggests sex is associated with ROM and patient experienced shoulder function after RTSA.*

#### Patient height

Two studies with low to moderate risk of bias evaluated the influence of patient height on shoulder function (ROM) [21] and patient experienced shoulder function (ASES- and SST score) [18, 21] (Table 3). The results of both studies showed that greater patient height is associated with better postoperative outcomes [18, 21]. Friedman et al. showed that greater patient height is associated with better postoperative ASES score and ROM (measured by active external and internal rotation) [21]. *According to our evidence synthesis, there is low quality evidence suggesting that greater patient height is associated with better ROM and moderate quality evidence for an association with better patient experienced shoulder function after RTSA.*

#### Dominant side

Two studies with low to moderate risk of bias evaluated the influence of surgery on the dominant arm on shoulder function (ROM) [17] and patient experienced shoulder function (SST score) [18]. Both studies showed that surgery on the dominant arm is associated with better postoperative function: higher ROM (measured by active forward flexion) and higher SST scores (Table 3) [17, 18]. Additionally, the data revealed that patients who had an operation on their dominant side had a greater success rate and faster recovery than patients with an operation on their non-dominant side [17, 18]. *According to our evidence synthesis, there is very low quality evidence suggesting that surgery on the dominant hand is associated with better postoperative ROM and better patient experienced shoulder function after RTSA.*

### Disorders

#### Diagnoses

Three studies with low to moderate risk of bias evaluated the influence of the preoperative diagnosis on patient experienced shoulder function (ASES score) [3, 18, 20]. Two studies showed that the presence of rotator cuff arthropathy was predictive for better postoperative ASES score compared to osteoarthritis (Table 3) [3, 18]. One study showed that the presence of rotator cuff arthropathy was associated with low pre-to-postoperative ASES score improvement (the bottom 30th percentile of improvement), which indicates a low degree of improvement [20]. *There is very low quality evidence for the association of diagnosis (rotator cuff arthropathy) with better postoperative patient experienced shoulder function after RTSA.*

#### Prior shoulder surgery

Two studies with low risk of bias evaluated the influence of previous shoulder surgery on shoulder function (ROM) [21] and patient experienced shoulder function (ASES score) [20, 21]. Both studies showed that prior shoulder surgery is associated with lower postoperative function: decreased ROM (measured by forward flexion) and lower ASES scores (Table 3), resulting in lower success rates [20, 21]. *According to our evidence synthesis, there is low quality evidence suggesting that no prior shoulder surgery is associated with better patient experienced shoulder function and better ROM after RTSA.*

#### Comorbidities

Two studies with low to moderate risk of bias evaluated the influence of comorbidities on shoulder function (ROM) [21] and patient experienced shoulder function (ASES score) [3]. One study showed that having more comorbidities (such as hypertension, diabetes or depression) was associated with poorer postoperative patient experienced shoulder function [3]. Meanwhile, the other study found an association between hypertension and postoperative ROM, but not with other comorbidities (Table 3) [21]. In this study, the presence of hypertension was associated with better postoperative forward flexion. *According to our evidence synthesis, there is low quality evidence for the association of comorbidities with postoperative ROM and very low quality evidence suggesting that comorbidities are associated with patient experienced shoulder function after RTSA.*

### Function & activity

#### Preoperative ASES score

Four studies with low to moderate risk of bias evaluated the influence of the preoperative ASES score on shoulder function (ROM) [21] and patient experienced shoulder function (ASES score) after RTSA [3, 18, 20, 21]. Two studies revealed that a better preoperative ASES score was associated with poorer postoperative improvement (measured using the ASES score) [3, 20], while two studies revealed that a better preoperative ASES score was associated with better postoperative ASES score [18, 21] (Table 3). Additionally, one study showed that a better preoperative ASES score was associated with better postoperative active internal rotation, but worse postoperative active external rotation [21]. *According to our evidence synthesis, there is low quality evidence suggesting that higher preoperative ASES score is associated with better ROM and patient experienced shoulder function after RTSA.*

#### Preoperative ROM

Three studies with low to moderate risk of bias evaluated the influence of the preoperative ROM on shoulder function (ROM) [16, 17, 21] and patient experienced shoulder function (ASES score) [21]. All studies showed that greater preoperative ROM was associated with greater postoperative ROM [16, 17, 21] measured by the degree of forward flexion, abduction and external rotation (Table 3). Though, Friedman et al. revealed in their study that greater preoperative abduction leads to lower postoperative forward flexion, but better postoperative abduction [21]. Besides, Friedman et al. showed that greater preoperative ROM (measured by external rotation and forward flexion) was associated with better postoperative ASES score [21]. *According to our evidence synthesis, there is moderate quality evidence suggesting that better preoperative ROM is associated with better ROM, and very low quality evidence for better preoperative ROM being associated with better patient experienced shoulder function after RTSA.*

#### Preoperative VAS score

Three studies with low to moderate risk of bias evaluated the influence of preoperative pain (VAS score and opioid use) on shoulder function (ROM) [21], patient experienced shoulder function (ASES- and SST scores) [18, 20] and pain (VAS score) [21]. One study showed that a higher preoperative VAS score was associated with better postoperative ASES- and SST scores [18]. Another study showed that greater preoperative opioid use was associated with lower postoperative ASES score and less improvement (the bottom 30th percentile of improvement) [20] (Table 3). Additionally, one study showed that a higher preoperative VAS score was associated with better postoperative VAS score (indicating less postoperative pain), but also with a lower postoperative ROM (measured by forward flexion) [21]. A*ccording to our evidence synthesis, there is very low quality evidence for the association of preoperative pain with patient experienced shoulder function, and low quality evidence suggesting that a higher preoperative VAS score is associated with better ROM after RTSA.*

#### Results GRADE

The GRADE PH was used to assess the overall quality of evidence of the included studies. The full results can be found in Appendix 2. The results of the overall quality of evidence are summarized in Table 4.

**Table 4:** Overall quality of evidence

Outcome	Predictors	Level of evidence
**Shoulder function**	Age	Very low
	Sex	Very low
	Height	Moderate
	Dominant arm	Very low
	Diagnosis	Very low
	Prior shoulder surgery	Low
	Preoperative ASES	Low
	Preoperative ROM	Very low
	Preoperative pain	Very low
	Opioid use	Very low
**ROM**	Age	Low
	Sex	Low
	Height	Low
	Dominant arm	Very low
	Prior shoulder surgery	Low
	Comorbidities	Low
	Preoperative ASES	Low
	Preoperative ROM	Moderate
	Preoperative pain	Low
**Infection rate**	Age	Very low
**Failure**	Sex	Very low
	Diagnosis	Very low
	Comorbidities	Very low
	Preoperative ASES	Very low
**Revision**	Sex	Low
**Satisfaction**	Sex	Very low

## Discussion

Eleven studies reported on preoperative predictive factors for postoperative outcomes after RTSA. Based on the evidence synthesis, we found moderate-quality evidence indicating that greater height predicts better postoperative shoulder function, and greater preoperative range of motion (ROM) predicts increased postoperative ROM. However, for all other predictive factors the quality of evidence was low or very low. The factors with moderate-quality evidence should be considered in the preoperative decision making for a RTSA.

Muscular strength restoration relies on the restoration of muscle length. Deltoid and infraspinatus length are known to be variable but are highly correlated with patient length [24]. Patients with greater height benefit from a larger lever arm, leading to improved range of motion and enhanced function after RTSA. In patients with greater height the joint is to be expected to be larger, accommodating greater motion. This also allows for the insertion of larger glenospheres, further contributing to the observed influence of height on range of motion.

Better preoperative ROM result in better ROM after RTSA. Collin et al. elucidated several reasons why poor preoperative ROM may be associated with recovery of ROM following RTSA [17]. Poor preoperative active deltoid function suggest significant functional compromise, potentially indicating chronic deltoid de-conditioning and a lack of a functional rotator cuff. Mizuno et al. previously noted that patients treated with an RTSA for primary glenohumeral arthritis and an intact rotator cuff demonstrated improved ROM compared to patients with rotator cuff arthropathy [25], highlighting the importance of a functional rotator cuff. Secondly, chronic poor ROM can leads to cortical adaptation [17]. Meaning lack of use in the daily life of a limb may, with time, remodel the brain and contribute to persistent deltoid weakness after RTSA. Initiating exercise therapy preoperatively in frail patients may reduce cortical adaptation, as suggested by previous research [26]. These findings are in line with literature on other orthopedic surgery populations, where ‘fitter’ patients tend to achieve better outcomes [27]. Importantly, this factor is modifiable before surgery. There is a growing body of evidence that preoperative education and exercise (prehabilitation), can increase the physiological reserve, physical capacity and ROM of patients before surgery, aiding postoperative (functional) recovery after major joint replacement surgery [28, 29]. In the case of RTSA, optimizing deltoid functioning preoperatively may contribute to improving postoperative range of motion.

The predictive value of age has been established for numerous orthopedic procedures, such as total hip and knee arthroplasty, and has revealed to be associated with various postoperative outcomes (such as lower ROM and lower Oxford score) [1]. Although only very low to low quality evidence was found in this review. The population of included studies mainly consisted of patients aged < 70 years, and no stratified analysis was performed. This may explain why no association between age and functioning or ROM was found in our review. In real-world practice the age variability of patients undergoing RSA is much larger, and so the true strength of the association between age and outcomes after RSA may differ.

In the Netherlands in 2020, it was reported that 12 to 16% of patients who underwent non-arthroplasty shoulder surgery ultimately required a shoulder arthroplasty procedure (LROI). Studies have indicated that patients undergoing total knee arthroplasty are at increased risks of postoperative complications if they had prior arthroscopic knee surgery [30]. The high prevalence of previous shoulder surgery, but limited quality available evidence highlight the need for an improved understanding of the association with postoperative outcomes after RTSA.

### Limitations

Most studies did not report on which operative techniques were used and included in the different studies. If different surgical procedures were indeed included, this could have had an important influence on the results, leading to high heterogeneity in the included population and surgical techniques. Conversely, the use of studies from different countries and healthcare settings improves the generalizability of our findings. Among the eleven included studies, four were prospective and seven retrospective. In general findings of retrospective cohort studies are less reliable than those of prospective studies.

For most predictive factors the quality of evidence was low or very low, meaning there is little certainty in the estimates and new studies are likely to influence the findings. A strength of our study is the large sample size of the total population included in the systematic review. The sample size varied from 137 to 1332 (mean 424; median 198) across the studies. The review process was limited because it relied on a limited number of evidence databases and did not consider grey literature. Moreover, due to the heterogeneity in the included studies, a meta-analysis was not feasible.

### Recommendation

Predicting the outcome of RTSA for individual patients is challenging, as prognoses vary substantially between patients. An accurate prediction model may contribute to objectifying an individual’s prognosis, identify risk factors and select the most beneficial treatment for each patient. For such a model to be developed, predictive factors capable of predicting postoperative outcomes must be identified. The quality of most identified predictive factors was weak, further high quality research is necessary to identify predictive factors. The grading of the evidence was mostly affected by the indirectness of evidence and publication bias. For almost all predictive factors only singular phase I studies were available, which are vulnerable to type I errors and publication bias. To improve the quality of evidence, phase II or III studie exploring the underlying mechanisms of predictive factors with the outcomes should be conducted [14].

## Conclusion

Our study analyzed which preoperative factors were predictive for multiple postoperative outcomes after RTSA. Overall there is low quality evidence on predictive factors for postoperative outcomes after RTSA. Based on moderate evidence only two factors could be considered in clinical practice: preoperative ROM and height. These predictors should be taken into account when counseling patients regarding RTSA and to establish more accurate patient specific expectations prior to surgery.

### Supplementary Information

Additional file 1: Appendix 1. Search strategy. 

Additional file 2: Appendix 2. GRADE PH table.

Additional file 3: Appendix 3. PRISMA checklist.

## Data Availability

The datasets used and/or analysed during the current study are available from the corresponding author on reasonable request.
