# Supplementary material for: Predictive factors for postoperative outcomes after reverse shoulder arthroplasty: a systematic review
Source: BMC Musculoskelet Disord. 2024 Jun 4;25:439. doi: 10.1186/s12891-024-07500-3 (PMC11151553; doi:10.1186/s12891-024-07500-3)

## **Appendix**

**Appendix 1: Search strategy**

((((Treatment outcome[MeSH Terms]) OR (Prognosis[MeSH Terms])) OR (Prediction)) AND ((Shoulder[MeSH Terms]) OR (Shoulder joint[MeSH Terms]))) AND (((((((((((Arthroplasty[MeSH Terms]) OR (Arthroplasty, replacement[MeSH Terms])) OR (Arthroplasty, replacement, shoulder[MeSH Terms])) OR (Prostheses and implants[MeSH Terms])) OR (Joint prosthesis[MeSH Terms])) OR (implantation, joint prosthesis[MeSH Terms])) OR (Shoulder prosthesis[MeSH Terms])) OR (Reverse shoulder arthroplasty)) OR (Reverse shoulder replacement)) OR (Reversed shoulder arthroplasty)) OR (Reversed shoulder replacement))


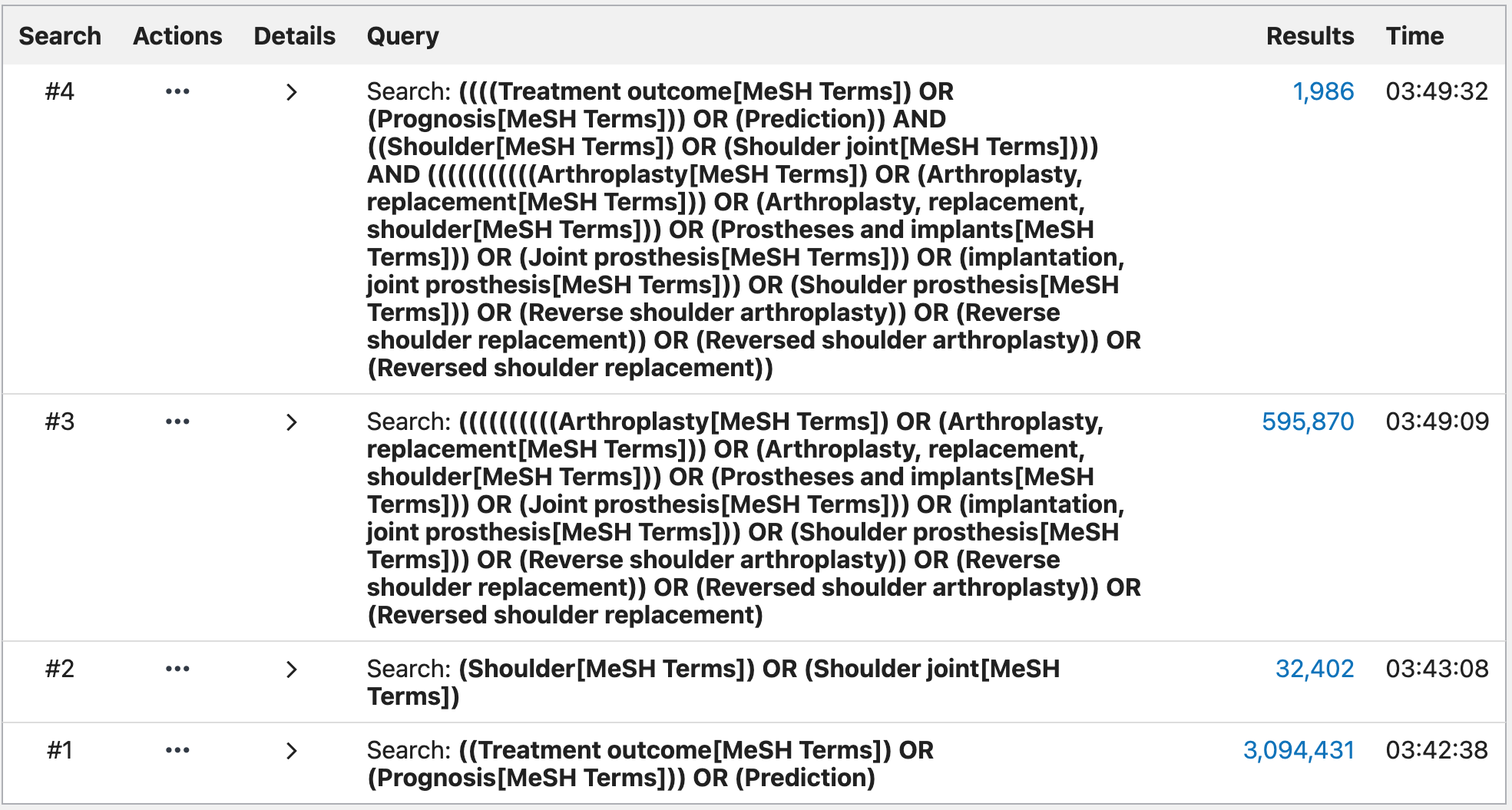

Supplement: Supplementary file 1 — Additional file 1: Appendix 1. Search strategy. [file 12891_2024_7500_MOESM1_ESM.docx]
