# Supplementary material for: Predictive factors for postoperative outcomes after reverse shoulder arthroplasty: a systematic review
Source: BMC Musculoskelet Disord. 2024 Jun 4;25:439. doi: 10.1186/s12891-024-07500-3 (PMC11151553; doi:10.1186/s12891-024-07500-3)
Supplement: Supplementary file 2 — Additional file 2: Appendix 2. GRADE PH table. [file 12891_2024_7500_MOESM2_ESM.docx]

Table - GRADE PH

| Outcome: shoulder functioning measured with ASES, SST or SPADI | | | | | | | | | | | | | | | | | | |
| --- | --- | --- | --- | --- | --- | --- | --- | --- | --- | --- | --- | --- | --- | --- | --- | --- | --- | --- |
|  |  |  |  | Univariate | | | Multivariate | | | GRADE factors | | | | | | |  |  |
| Potential prognostic factors identified | Number of participants | Number of studies | Number of cohorts | + | 0 | - | + | 0 | - | Phase | Study limitations | Inconsistency | Indirectness | Imprecision | Publication bias | Moderate/large effect size | Dose effect | Overall quality |
| Age | 660 | 1 | 1 | 0 | 0 | 0 | 0 | 0 | 2 | 1 | - | Na | - | + | - | - | Na | Very low |
| Sex | 198 | 1 | 1 | 1? | 0 | 1? | 0 | 0 | 0 | 1 | - | na | - | + | - | - | na | Very low |
| Height | 1530 | 2 | 2 | 3 | 0 | 0 | 0 | 0 | 0 | 1 | +- | Na | + | + | - | + | Na | Moderate |
| Dominant arm | 198 | 1 | 1 | 1 | 0 | 0 | 1 | 0 | 0 | 1 | - | na | - | + | - | + | na | Very low |
| Diagnosis | 335 | 2 | 2 | 1 | 0 | 0 | 1 | 0 | 1 | 1 | - | Na | - | + | - | - | Na | Very low |
| Prior shoulder surgery | 1469 | 2 | 2 | 0 | 0 | 1 | 0 | 0 | 1 | 1 | + | Na | + | + | - | - | Na | Low |
| Preoperative ASES | 1667 | 3 | 2 | 3 | 0 | 0 | 0 | 0 | 0 | 1 | +- | Na | + | + | - | - | Na | Low |
| Preoperative ROM | 1332 | 1 | 1 | 1 | 0 | 0 | 0 | 0 | 0 | 1 | + | Na | - | + | - | - | Na | Very low |
| Preopeartive pain | 198 | 1 | 1 | 0 | 0 | 2 | 0 | 0 | 0 | 1 | - | Na | - | + | - | - | Na | Very low |
| Opioid use | 137 | 1 | 1 | 0 | 0 | 0 | 2 | 0 | 0 | 1 | + | Na | - | - | - | - | Na | Very low |

| Outcome: ROM | | | | | | | | | | | | | | | | | | |
| --- | --- | --- | --- | --- | --- | --- | --- | --- | --- | --- | --- | --- | --- | --- | --- | --- | --- | --- |
|  |  |  |  | Univariate | | | Multivariate | | | GRADE factors | | | | | | |  |  |
| Potential prognostic factors identified | Number of participants | Number of studies | Number of cohorts | + | 0 | - | + | 0 | - | Phase | Study limitations | Inconsistency | Indirectness | Imprecision | Publication bias | Moderate/large effect size | Dose effect | Overall quality |
| Age | 1332 | 1 | 1 |  |  | 1 |  |  |  | 1 | + | Na | - | + | - | + | Na | Low |
| Sex | 1332 | 1 | 1 | 1 |  |  |  |  |  | 1 | + | Na | - | + | - | + | Na | Low |
| Height | 1332 | 1 | 1 | 1 |  |  |  |  |  | 1 | + | Na | - | + | - | + | Na | Low |
| Dominant arm | 101 | 1 | 1 |  |  |  | 1 |  |  | 1 | + | Na | - | - | - | + | Na | Very low |
| Prior shoulder surgery | 1332 | 1 | 1 |  |  | 1 |  |  |  | 1 | + | Na | - | + | - | + | Na | Low |
| Comorbidities | 1332 | 1 | 1 | 1 |  |  |  |  |  | 1 | + | Na | - | + | - | + | Na | Low |
| Preoperative ASES | 1332 | 1 | 1 | 1 |  |  |  |  |  | 1 | + | Na | - | + | - | + | Na | Low |
| Preoperative ROM | 1973 | 3 | 3 | 1 |  |  | 2 |  |  | 1 | +- | Na | + | + | - | + | Na | Moderate |
| Preopeartive pain | 1332 | 1 | 1 | 1 |  |  |  |  |  | 1 | + | Na | - | + | - | + | Na | Low |

| Outcome: infection rate | | | | | | | | | | | | | | | | | | |
| --- | --- | --- | --- | --- | --- | --- | --- | --- | --- | --- | --- | --- | --- | --- | --- | --- | --- | --- |
|  |  |  |  | Univariate | | | Multivariate | | | GRADE factors | | | | | | |  |  |
| Potential prognostic factors identified | Number of participants | Number of studies | Number of cohorts | + | 0 | - | + | 0 | - | Phase | Study limitations | Inconsistency | Indirectness | Imprecision | Publication bias | Moderate/large effect size | Dose effect | Overall quality |
| Age | 68 | 1 | 1 | 1 |  |  | 1 |  |  | 1 | + | Na | - | - | - | + | Na | Very low |

| Outcome: failure | | | | | | | | | | | | | | | | | | |
| --- | --- | --- | --- | --- | --- | --- | --- | --- | --- | --- | --- | --- | --- | --- | --- | --- | --- | --- |
|  |  |  |  | Univariate | | | Multivariate | | | GRADE factors | | | | | | |  |  |
| Potential prognostic factors identified | Number of participants | Number of studies | Number of cohorts | + | 0 | - | + | 0 | - | Phase | Study limitations | Inconsistency | Indirectness | Imprecision | Publication bias | Moderate/large effect size | Dose effect | Overall quality |
| Sex | 1699 | 1 | 1 | 1 |  |  |  |  |  | 1 | - | Na | - | + | - | + | Na | Very low |
| Diagnosis | 1699 | 1 | 1 | 1 |  |  |  |  |  | 1 | - | Na | - | + | - | + | Na | Very low |
| Comorbidities | 1699 | 1 | 1 |  |  | 1 |  |  |  | 1 | - | Na | - | + | - | + | Na | Very low |
| Preoperative ASES | 1699 | 1 | 1 |  |  | 1 |  |  |  | 1 | - | Na | - | + | - | + | Na | Very low |

| Outcome:  revision | | | | | | | | | | | | | | | | | | |
| --- | --- | --- | --- | --- | --- | --- | --- | --- | --- | --- | --- | --- | --- | --- | --- | --- | --- | --- |
|  |  |  |  | Univariate | | | Multivariate | | | GRADE factors | | | | | | |  |  |
| Potential prognostic factors identified | Number of participants | Number of studies | Number of cohorts | + | 0 | - | + | 0 | - | Phase | Study limitations | Inconsistency | Indirectness | Imprecision | Publication bias | Moderate/large effect size | Dose effect | Overall quality |
| Sex | 150 | 1 | 1 | 1 |  |  |  |  |  | 1 | + | Na | - | + | - | + | Na | Low |

| Outcome:  satisfaction | | | | | | | | | | | | | | | | | | |
| --- | --- | --- | --- | --- | --- | --- | --- | --- | --- | --- | --- | --- | --- | --- | --- | --- | --- | --- |
|  |  |  |  | Univariate | | | Multivariate | | | GRADE factors | | | | | | |  |  |
| Potential prognostic factors identified | Number of participants | Number of studies | Number of cohorts | + | 0 | - | + | 0 | - | Phase | Study limitations | Inconsistency | Indirectness | Imprecision | Publication bias | Moderate/large effect size | Dose effect | Overall quality |
| Sex | 161 | 1 | 1 |  |  |  |  |  | 1 | 1 | + | Na | - | - | - | + | Na | Very low |
